# Regionally selective cardiovascular responses to adenosine A_2A_ and A_2B_ receptor activation

**DOI:** 10.1096/fj.202101945R

**Published:** 2022-03-01

**Authors:** Samantha L. Cooper, Edward S. Wragg, Patrizia Pannucci, Mark Soave, Stephen J. Hill, Jeanette Woolard

**Affiliations:** ^1^ Division of Physiology, Pharmacology and Neuroscience School of Life Sciences University of Nottingham Nottingham UK; ^2^ Centre of Membrane Proteins and Receptors University of Birmingham and University of Nottingham Midlands UK

**Keywords:** A_2_ receptor activation, adenosine, Doppler flow, hemodynamics

## Abstract

Adenosine is a local mediator that regulates changes in the cardiovascular system via activation of four G protein‐coupled receptors (A_1_, A_2A_, A_2B_, A_3_). Here, we have investigated the effect of A_2A_ and A_2B_‐selective agonists on vasodilatation in three distinct vascular beds of the rat cardiovascular system. NanoBRET ligand binding studies were used to confirm receptor selectivity. The regional hemodynamic effects of adenosine A_2A_ and A_2B_ selective agonists were investigated in conscious rats. Male Sprague‐Dawley rats (350–450 g) were chronically implanted with pulsed Doppler flow probes on the renal artery, mesenteric artery, and the descending abdominal aorta. Cardiovascular responses were measured following intravenous infusion (3 min for each dose) of the A_2A_‐selective agonist CGS 21680 (0.1, 0.3, 1 µg kg^−1^ min^−1^) or the A_2B_‐selective agonist BAY 60‐6583 (4,13.3, 40 µg kg^−1^ min^−1^) following predosing with the A_2A_‐selective antagonist SCH 58261 (0.1 or 1 mg kg^−1^ min^−1^), the A_2B_/A_2A_ antagonist PSB 1115 (10 mg kg^−1^ min^−1^) or vehicle. The A_2A_‐selective agonist CGS 21680 produced a striking increase in heart rate (HR) and hindquarters vascular conductance (VC) that was accompanied by a significant decrease in mean arterial pressure (MAP) in conscious rats. In marked contrast, the A_2B_‐selective agonist BAY 60‐6583 significantly increased HR and VC in the renal and mesenteric vascular beds, but not in the hindquarters. Taken together, these data indicate that A_2A_ and A_2B_ receptors are regionally selective in their regulation of vascular tone. These results suggest that the development of A_2B_ receptor agonists to induce vasodilatation in the kidney may provide a good therapeutic approach for the treatment of acute kidney injury.

**Abbreviations**
A_1_
: adenosine A_1_ receptor
A_2A_
: adenosine A_2A_ receptor
A_2B_
: adenosine A_2B_ receptor
A_3_
: adenosine A_3_ receptor
HR: heart rate
MAP: mean arterial pressure
NanoBRET: nano‐luciferase bioluminescence resonance energy transfer
Nluc: nano‐luciferase
VC: vascular conductance

## 1 INTRODUCTION

Adenosine is a purine nucleoside that is present in all cells in tightly regulated concentrations and plays an important protective and regenerative role in the heart and vasculature.^1^, ^2^, ^3^ The adenosine A_2A_ and A_2B_ receptors are two of the four known adenosine receptor subtypes.^1^, ^2^, ^3^ Both A_2A_ and A_2B_‐receptors couple to stimulatory G_s_‐proteins and mediate activation of adenylyl cyclase and the formation of intracellular cyclic AMP.^1^, ^2^, ^3^ The A_2A_ receptor is highly sensitive to adenosine and is widely distributed in the brain, heart, lung, immune cells and the vasculature.^3^, ^4^ The A_2B_ receptor has a low affinity for adenosine and ubiquitous tissue distribution.^3^ In the cardiovascular system, A_2B_ receptors are highly expressed in fibroblasts, vascular beds, and myocardial cells.^3^, ^5^ Both receptors promote vasodilatation and play an important role in cardiovascular physiology.^3^, ^5^, ^6^ As a result, A_2A_ and A_2B_ receptors have been suggested as potential targets for the treatment of hypertension and cardiovascular diseases.^5^, ^7^, ^8^

Systemic administration of non‐selective adenosine A_2A_/A_2B_ receptor agonists produce hypotension that is believed to be a direct consequence of peripheral vasodilatation induced by A_2A_/A_2B_‐receptor activation or due to an A_2A_/A_2B_‐mediated increase in renal blood flow and subsequent natriuresis, which reduces blood pressure.^5^, ^9^, ^10^, ^11^, ^12^ Corroborating this, the selective A_2A_ adenosine receptor agonist, CGS 21680,^2^, ^13^, ^14^ is a potent vasodilator that induces hypotension and a marked increase in heart rate (HR) that is consistent with a reflex increase in sympathetic nervous activity in the rat.^15^ A role for A_2A_ and A_2B_ receptors in vasodilatation in response to adenosine analogs has also come from studies with genetically modified mice lacking the A_2A_ receptor.^16^, ^17^ A_2A_ receptor knock‐out mice are also known to be hypertensive.^7^ Furthermore, studies using both A_2A_ and A_2B_ receptor knock‐out mice have also confirmed a role for both receptors in coronary vasodilatation in a Langendorff perfused heart model.^18^

Although A_2B_ receptors have been shown to induce vasodilatation in ex vivo models,^11^, ^18^ there is limited knowledge of the hemodynamic effects of A_2B_ selective agonists in vivo. We have previously shown that adenosine can elicit vasodilatation in the renal, mesenteric, and hindquarters’ vascular beds of conscious rats that is not attenuated by the selective A_1_‐receptor antagonist DPCPX and is likely to be mediated by A_2_ receptors.^19^ However, the relative extent to which each A_2_ receptor contributes to vasodilatation in different vascular beds has not been investigated previously. In the present study, we have used selective agonists for the A_2A_ (CGS 21680)^14^ and A_2B_ (BAY 60‐6583)^14^, ^20^ receptors to investigate the hemodynamic effects of A_2A_ and A_2B_ receptor stimulation in conscious, freely‐moving rats and compared the effect of each agonist on vascular flow in three different vascular beds (mesenteric, renal, and hindquarters) simultaneously.

## 2 MATERIALS AND METHODS

### 2.1 Drugs, chemical reagents and other material

4‐[2‐[[6‐Amino‐9‐(*N*‐ethyl‐β‐D‐ribofuranuronamidosyl)‐9*H*‐purin‐2‐yl]amino]ethyl]benzenepropanoic acid hydrochloride (CGS 21680 hydrochloride) (Cat#1063), 2‐(2‐Furanyl)‐7‐(2‐phenylethyl)‐7*H*‐pyrazolo[4,3‐*e*][1,2,4]triazolo[1,5‐*c*]pyrimidin‐5‐amine (SCH 58261) (Cat#2270), 2‐[[6‐Amino‐3,5‐dicyano‐4‐[4‐(cyclopropylmethoxy)phenyl]‐2‐pyridinyl]thio]‐acetamide (BAY 60‐6583) (Cat#4472), 4‐(2,3,6,7‐Tetrahydro‐2,6‐dioxo‐1‐propyl‐1*H*‐purin‐8‐yl)‐benzenesulfonic acid (PSB 1115) (Cat#2009), 8‐[4‐[4‐(4‐Chlorophenzyl)piperazide‐1‐sulfonyl)phenyl]]‐1‐propylxanthine (PSB 603) (Cat#3198), 1‐(6‐Amino‐9H‐purin‐9‐yl)‐1‐deoxy‐N‐ethyl‐β‐D‐ribofuranuronamide (NECA) (Cat#1691), and (±)‐(*R*,*R*)‐*N*‐[2‐Hydroxy‐5‐[1‐hydroxy‐2‐[[2‐(4‐methoxyphenyl)‐1‐methylethyl]amino]ethyl]phenyl] formamide hemifumarate (formoterol) (Cat#1448) were purchased from Tocris Bioscience (Bristol, UK). Dimethyl Sulfoxide (DMSO) (Cat#D5879) and Bovine Serum Albumin (BSA) (Cat#A7030) were purchased from Sigma‐Aldrich (Gillingham, UK). The fluorescent ligand, CA200645, was purchased from Hello Bio, (Bristol, UK) (Cat#HB7812). Exclusively for the in vitro experiments described, the ligands were dissolved in DMSO from powdered stocks to 10^−2^ M prior to the commencement of the experiment.

Fentanyl citrate was purchased from Martindale Pharmaceuticals (Essex, UK). Medetomidine Hydrochloride (Sedastart), Atipamezole Hydrochloride (Sedastop), and Buprenorphine (Buprecare) were purchased from Animalcare Ltd. (York, UK). Meloxicam (Metacam) was purchased from Boehringer Ingelheim Animal Health UK (Berkshire, UK). Pentobarbitone (Euthatal) was purchased from Alstoe Animal Health, (York, UK). Tween 80 and propylene glycol were purchased from Sigma‐Aldrich (Gillingham, UK).

### 2.2 Constructs, cell lines and cell culture

Rat A_2A_ and A_2B_ receptor cDNA was obtained from Missouri S&T cDNA Resource Center (www.cdna.org) in a pcDNA3.1 expression vector. N‐terminal Nano luciferase (Nluc)‐labeled rat adenosine A_2A_ and A_2B_ receptor constructs (Nluc‐A2AR and Nluc‐A2BR, respectively) were generated in frame with the full length Nluc incorporating a 5‐HT3A membrane localization signal sequence in pcDNA3.1 (pcDNA3.1 sig‐NL)^21^ as described previously. The full‐length sequence of the rat A_2A_ or A_2B_ adenosine receptor was first mutated to remove internal BamHI (A_2A_) and XhoI (A_2B_) restriction sites with site‐directed mutagenesis using the following primers:

Rat A_2A_

Fwd 5′: CATCTACGCCTACAGAATCCGGGAGTTCCGC;

Rev 5′:‐GCGGAACTCCCGGATTCTGTAGGCGTAGATG;

Rat A_2B_

Fwd 5′:‐GCGGTGGGAGCCTCTAGTGCTTTACAGAC;

Rev 5′:‐GTCTGTAAAGCACTAGAGGCTCCCACCGC.

Linear rat A_2A_ or A_2B_ cDNA fragments were generated via PCR with the following oligonucleotide primers (Rat A_2A_ Fwd 5′:‐AGAGGATCCCTGGGCTCCTC, Rat A_2A_ Rev 5′:‐CCTCGAGTCAGGAAGGGGCAAAC; Rat A_2B_ Fwd 5′:‐AGAGGATCCCTGCAGCTAGAG, Rev 5′:‐CCCTCGAGTCACAAGCTCAG) to insert BamHI and XhoI restriction sites and to mutate the initial methionine to a leucine. These linear fragments were subsequently ligated into pcDNA3.1 sig‐NL using the BamHI and XhoI restriction enzymes resulting in a fusion protein containing a Gly‐Ser linker between the Nluc open reading frame (ORF) and the rat A_2A_ or A_2B_ ORF.

HEK293T cells (CRL‐3216) were obtained from ATCC (Virginia, USA). A clonal HEK 293 cell line stably expressing the cAMP GloSensor (20F) biosensor (HEK293G)^14^, ^22^ was obtained from Promega (Madison, WI, USA).

### 2.3 Cultured cells

HEK293T cells were maintained in Dulbecco's Modified Eagle Medium (DMEM) supplemented with 2 mM L‐glutamine and 10% Fetal Calf Serum (FCS) incubated at 37ºC/5% CO_2_ and grown to 70%–80% confluency in T75 flasks.

72 h before the experiment, cells were incubated with trypsin (0.25%) and dislodged from the flask surface by gentle shaking. Next, the dislodged cells were resuspended in DMEM and centrifuged for 3 min at 1000 g. Following this, the pellet of cells was resuspended in 10 ml of DMEM. Finally, a 100 mm × 20 mm Style Dish (Corning) was seeded to a density of 2.0 × 10^6^ cells per dish in 11 ml of DMEM.

48 h before the experiment, cells were transiently transfected with rat Nluc‐A_2A_R or rat Nluc‐A_2B_R cDNA using FuGENE HD (Promega). First, 6.0 µg of the appropriate cDNA stock was diluted to 0.020 µg µl^–1^ in OptiMEM, before 18 μl of FuGENE (1:3 cDNA/FuGENE ratio) was added to the cDNA mixture, gently flicked to mix, and incubated for 10 min at room temperature to allow the FuGENE to form a complex with the cDNA. After that time, the entire mixture was added to 11 ml of fresh DMEM. The media on the cells was then aspirated off and replaced with the transfection mixture.

24 h before the experiment, cells were removed from the dish and centrifuged as described above. Then, the pellet of cells was resuspended in 10 ml of DMEM, and the resulting Nluc‐tagged HEK293T cells seeded at a density of 30 000 cells per well in clear‐bottomed white‐walled 96‐well microplates (Thermo Scientific), which were pretreated with poly‐D‐lysine.

HEK293G cells were maintained and seeded into 100 mm × 20 mm style dishes (Corning) as described above for HEK293T cells, with the exception that the cells were seeded at a higher density of 3.0 × 10^6^ cells per dish to account for a marginally slower rate of growth. 48 h before the experiment, the media on the cells was aspirated off and replaced with fresh media. 24 h before the experiment, the cells were plated into clear‐bottomed white‐walled 96‐well microplates (Thermo Scientific) as described above for HEK293T cells.

### 2.4 NanoBRET rat NL‐A_2A_R and NL‐A_2B_R ligand‐binding assays

Fluorescent antagonist saturation and competition‐binding assays were performed on transiently transfected HEK293T cells expressing Rat Nluc‐A_2A_R or Nluc‐A_2B_R. The following day, DMEM was replaced with a HEPES‐buffered saline solution (HBSS; 147 mM NaCl, 5 mM KCl, 1.3 mM CaCl_2_, 1 mM MgSO_4_, 10 mM HEPES, 2 mM sodium pyruvate, 1.43 mM NaHCO_3_, 10 mM d‐glucose, pH 7.45, supplemented with 0.1% BSA), and the required concentrations of fluorescent ligand (CA200645) and competing ligands added to the 96‐well microplate. For saturation binding assays, concentrations of 0–500 nM of CA200645 were used in the presence or absence of a receptor selective antagonist (for A_2A_: 10 µM of SCH 58261 and A_2B_: 10 µM of PSB 603). For competition binding assays, 50 nM of CA200645 was used in the presence of increasing concentrations of unlabeled ligands. At the A_2A_ receptor, increasing concentrations of SCH 58261 (0.01 nM–10 µM), PSB 603 (0.1 nM–100 µM), PSB 1115 (0.1 nM–100 µM), CGS 216880 (0.1 nM–100 µM), BAY 60–6583 (0.1 nM–100 µM), and NECA (100 µM–0.1 nM) were used, and at the A_2B_ receptor, concentrations of SCH 58261 (0.1 nM–100 µM), PSB 1115 (0.1 nM–100 µM), PSB 603 (0.01 nM–10 µM), BAY 60–6583 (0.1 nM–100 µM), CGS 216880 (0.1 nM–100 µM), and NECA (0.1 nM–100 µM) were used, with the DMSO concentration in all wells equalized to a final in‐well DMSO concentration of 1%. The cells were then incubated for 2 h at 37°C. Furimazine (Promega), the Nluc substrate, was then added to each well to give a final concentration of 10 µM. The cells were incubated for a further 5 min at 37°C. A PHERAstar FS plate reader (BMG Labtech) was used to measure the resulting BRET using filtered light emissions at 460 nm (80 nm bandpass) and >610 nm (longpass) at 37°C. The ratio between >610 nm emission and the 460 nm emission provided the raw BRET data for each experiment.

### 2.5 GloSensor cyclic AMP assay

The GloSensor assay was carried out as per the manufacturer's instructions (Promega, Madison, WI, USA). Briefly, media was aspirated and cells incubated in 50 µl of the HEPES‐buffered saline solution (HBSS) described above for the NanoBRET rat ligand‐binding assays (2.4), containing 3% GloSensor cAMP reagent (Promega), for 2 h at a final experimental temperature of 37°C. Luminescence was measured on the PHERAstar FS plate reader continuously over 60 min, averaging 1 read per well every 1.0 min, following the addition of 50 µl HBSS in the presence or absence of NECA (100 µM), BAY 60‐6583 (0.1 nM–100 µM) or CGS 21680 (0.1 nM–100 µM), and SCH 58261 (10 µM) or formoterol (10 µM) with each ligand in isolation or in combination, with the DMSO concentration in all wells equalized to a final in‐well DMSO concentration of 1%. When used, 10 µM PSB 603 was incubated for 2 h before the addition of other ligands.

### 2.6 Animals and surgery

Experiments were carried out on male Sprague‐Dawley rats (Charles River Laboratories, UK; weights 350 to 450 g). Animals were group‐housed in a temperature‐controlled (21–23°C) environment with a 12 h light‐dark cycle (lights on at 6:00 am) with free access to food (18% Protein Rodent Diet; Envigo, Madison WI, USA) and water for a minimum of 7 days prior to any surgical intervention. All procedures were approved by the University of Nottingham Animal Welfare and Ethical Review Board and were performed in line with the Animals (Scientific Procedures) Act (1986), under the UK Home Office approved Project Licence and the Personal License authority. Forty‐two rats were used for this study, and all animal studies are reported in compliance with the ARRIVE guidelines^23^ and the editorial on reporting animal studies.^24^

### 2.7 Surgery

Under general anesthesia (fentanyl and medetomidine, 300 μg kg^–1^ each, i.p., supplemented as required), miniature pulsed Doppler flow probes were implanted around the left renal and superior mesenteric arteries and the descending abdominal aorta (providing blood flow to the hindquarters) to monitor Doppler shift.^25^ The probe wires were secured to the abdominal wall and led subcutaneously to the posterior of the neck. The wires were then secured with suture and sterile tape to the nape of the neck. Reversal of anesthesia and postoperative analgesia was provided by atipamezole hydrochloride (1.0 mg kg^–1^, s.c.) and buprenorphine (30 µg kg^–1^, s.c.). A second dose of analgesia (buprenorphine 15 µg kg^–1^, s.c.) was given 4 h post‐surgery. Additional analgesia (meloxicam, 1.0 mg kg^–1^ day^–1^, s.c.) was given before the start of the surgical procedure, and also daily for a further three days post‐operation.

At least 10 days after probe implantation and after a satisfactory inspection from the Named Veterinary Surgeon, catheter implantation was performed under anesthesia (fentanyl and medetomidine, 300 μg kg^–1^ each, i.p., supplemented as required). The catheters were filled with heparinized saline (15 U ml^–1^) and were inserted into the distal abdominal aorta via the ventral caudal artery (positioned to monitor arterial blood pressure and HR). Three intravenous catheters were implanted into the right jugular vein for drug administration.^19^ All catheters were led subcutaneously to the nape of the neck.

The probe wires were released from the nape of the neck, soldered into a miniature plug (Omnetic connector corporation, USA) and mounted onto a custom‐designed harness worn by the rat. Secured to the harness was a spring that the catheters and probe wires ran through for protection. A counterbalanced pivot system supported this whole assembly to allow the free movement of the animal. Reversal of anesthetic and analgesia was administered (as described above). The arterial catheter was infused with heparinized (15 U ml^–1^) saline overnight to maintain potency.

Experiments began 24 h after surgery for catheter implantation, with animals fully conscious and unrestrained in home cages, with access to food and water *ad libitum*.

### 2.8 Cardiovascular recordings

During the cardiovascular monitoring periods, rats were connected to the customized data‐acquisition software, described below, via a tether system. Recordings were made for at least 30 min prior to the administration of any interventions and continuously for a minimum of 4 h thereafter. HR, arterial blood pressure, and renal, mesenteric, and hindquarters Doppler shifts were measured by a transducer amplifier (13‐4615‐50; Gould, Cleveland, OH, USA), a Doppler flowmeter (Crystal Biotech, Holliston, MA, USA), and a VF‐1 mainframe (pulse repetition frequency 125 kHz) fitted with high‐velocity (HVPD‐20) modules. These measurements were recorded by customized computer software (IdeeQ; Maastricht Instruments, Maastricht, The Netherlands). Raw data were sampled by IdeeQ every 2 ms, averaged, and stored to disk every cardiac cycle. Changes in renal vascular conductance (VC), mesenteric VC, and hindquarter VC were calculated from the changes in mean arterial pressure (MAP) and Doppler shift.

### 2.9 Experimental protocol

Experiments were run in 5 studies, each lasting 3 days; within each study was a contemporaneous vehicle control (5% propylene glycol, 2% Tween 80 in sterile saline). Experiments were run with treatment groups of 8 to 9 rats. We were unable to maintain the A_2B_ receptor antagonist PSB 603 in solution in the required propylene glycol, Tween 80 vehicle and so used the more water‐soluble PSB 1115 instead for in vivo studies.

#### 2.9.1 Study 1: The effect of A_2A_ antagonist SCH 58261 (0.1 mg kg^–1^) on the hemodynamic profile of A_2A_ agonists CGS 21680

Nine animals were used to assess the cardiovascular responses to CGS 21680 in the presence or absence of SCH 58261. After a period of baseline recordings, rats were randomized into two groups. **Group 1** received vehicle *via* intravenous bolus (0.1 ml provided over 5 s) on day 1 and a SCH 58261 (0.1 mg kg^–1^) intravenous bolus on day 3. **Group 2** received SCH 58261 (0.1 mg kg^–1^, 0.1 ml bolus, i.v.) on day 1 and vehicle (0.1 ml bolus, i.v.) on day 3. Approximately 10 min after the initial bolus of vehicle or SCH 58261, all groups received intravenous infusions (0.1 ml min^–1^) of CGS 21680 (0.1 (low), 0.3 (mid), and 1 (high) µg kg^−1^ min^−1^). Each dose was infused for 3 min. Hemodynamic recordings were made for a further 4 h following the completion of the CGS 21680 intravenous infusion period.

#### 2.9.2 Study 2: The effect of A_2A_ antagonist SCH 58261 (1 mg kg^–1^) on the hemodynamic profile of A_2A_ agonists CGS 21680

Eight animals were used to measure the cardiovascular responses to CGS 21680 in the presence or absence of a higher dose of SCH 58261. Following a period of baseline recording, rats were randomized into two groups. **Group 1** received vehicle (0.1 ml bolus, i.v.) on day 1 and SCH 58261 (1 mg kg^–1^ bolus, i.v.) on day 3. **Group 2** received a single 0.1 ml bolus of SCH 58261 (1 mg kg^–1^, i.v.) on day 1 and vehicle (bolus, 0.1 ml) on day 3. After 10 min all groups received intravenous infusions (0.1 ml min^–1^) of CGS 21680 (0.1 (low), 0.3 (mid), and 1 (high) µg kg^−1^ min^−1^). Each dose of CGS 21680 was given as a 3‐min infusion. Cardiovascular recordings were continued for a further 4 h after administration of CGS 21680.

#### 2.9.3 Study 3: The effect of A_2B_ antagonist PSB 1115 on the hemodynamic profile of A_2A_ agonists CGS 21680

Eight animals were used to assess the cardiovascular responses to CGS 21680 in the presence or absence of PSB 1115. Following a period of baseline recording, rats were randomized into two groups. **Group 1** received vehicle (0.1 ml bolus, i.v.) on day 1 and PSB 1115 (10 mg kg^−1^ bolus, i.v.) on day 3. **Group 2** received a single 0.1 ml bolus of PSB 1115 (10 mg kg^−1^, i.v.) on day 1 and vehicle (bolus, 0.1 ml) on day 3. After 10 min, all groups received intravenous infusions (0.1 ml min^–1^) of CGS 21680 (0.1 (low), 0.3 (mid), and 1 (high) μg kg^−1^ min^−1^). Each dose of CSS 21680 was given as a 3 min infusion. Cardiovascular recordings were continued for a further 4 h after administration of the CGS 21680.

#### 2.9.4 Study 4: The effect of A_2B_ antagonist PSB 1115 on the hemodynamic profile of A_2B_ agonists BAY 60‐6583

Eight animals were used to assess the cardiovascular responses to BAY 60‐6583 in the presence or absence of PSB 1115. Following a period of baseline recording, rats were randomized into 2 groups. **Group 1** received vehicle (0.1 ml bolus, i.v.) on day 1 and PSB 1115 (10 mg kg^−1^ bolus, i.v.) on day 3. **Group 2** received a single 0.1 ml bolus of PSB 1115 (10 mg kg^−1^, i.v.) on day 1 and vehicle (bolus, 0.1 ml) on day 3. After 10 min, all groups received intravenous infusions (0.1 ml min^–1^) of BAY 60‐6583 (4 (low), 13.3 (mid), and 40 (high) μg kg^−1^ min^−1^). Each dose of BAY 60‐6583 was given as a 3 min infusion. Cardiovascular recordings were continued for a further 4 h after administration of the BAY 60‐6583.

#### 2.9.5 Study 5: The effect of A_2A_ antagonist SCH 58261 (1 mg kg^–1^) on the hemodynamic profile of A_2B_ agonists BAY 60‐6583

Nine animals were used to assess the cardiovascular responses to BAY 60‐6583 in the presence or absence of SCH 58261. Following a period of baseline recording, rats were randomized into 2 groups. **Group 1** received vehicle (0.1 ml bolus, i.v.) on day 1 and SCH 58261 (1 mg kg^−1^ bolus, i.v.) on day 3. **Group 2** received a single 0.1 ml bolus of SCH 58261 (1 mg kg^‐1^, i.v.) on day 1 and vehicle (bolus, 0.1 ml) on day 3. After 10 min, all groups received intravenous infusions (0.1 ml min^–1^) of BAY 60‐6583 (0.1 (low), 0.3 (mid), and 1 (high) μg kg^−1^ min^−1^). Each dose of CSS 21680 was given as a 3 min infusion. Cardiovascular recordings were continued for a further 4 h after administration of the BAY 60‐6583.

### 2.10 Data analysis

All in vivo data were collected and analyzed using IdeeQ software (Maastricht Instrumates, Maastricht University, NL). For all experiments, time‐averaged data are shown as changes from baseline [HR (beats min^–1^); MAP (mmHg); VC (%)]. Statistical comparisons between groups of animals were performed on the integrated changes over specified time periods. A Friedman‘s test, which is a nonparametric, repeated‐measures analysis of variance, was used for within‐group comparisons, and a Wilcoxon rank‐sum test for integrated area under or above curve analysis was used for comparisons between groups. A Wilcoxon test was also performed for comparisons between groups at a specific time point. VCs were calculated from the MAP and Doppler shift (flow) data. A value of *p* < .05 was considered significant.

For all NanoBRET experiments, data were presented and analyzed using Prism 9.0 software (GraphPad software, San Diego, CA, USA). Saturation binding curves were fitted to determine total and nonspecific binding components of the fluorescent antagonist CA200645, using the following equation:

$$\text{BRET}\;\text{ratio}:\frac{B_{\text{max}}\times \left[B\right]}{\left[B\right]+K_{D}}+\left(M\times \left[B\right]\right)+C,$$

where *B*_max_ is the maximal level of specific binding, [*B*] is the concentration of fluorescent ligand in nM, *K_D_* is the equilibrium dissociation constant in nM, *M* is the slope of the linear nonspecific binding component, and *C* is the *y*‐axis intercept. The BRET ratio observed with increasing concentrations of each of the competing ligands was fitted using a one‐site sigmoidal response curve given by the following equation:

$$\%\;\text{uninhibited}\;\text{binding}=100‐\frac{(100\times \left[{A]}^{n}\right)}{\left({\left[A\right]}^{n}+{\text{IC}}_{50}^{n}\right)}+\text{NS},$$

where, [*A*] is the concentration of the competing drug, NS is the nonspecific binding, *n* is the Hill coefficient, and IC_50_ is the concentration of ligand required to inhibit 50% of the specific binding of the fluorescent ligand. The IC_50_ values obtained from the competition‐binding assays were used to calculate the *K_i_* of the unlabeled ligands using the Cheng–Prusoff equation,

$$K_{i}=\frac{{\text{IC}}_{50}}{1+\frac{\left[L\right]}{K_{D}}},$$

where [*L*] is the concentration of fluorescent ligand in nM, and *K_D_* is the dissociation constant of the fluorescent ligand in nM. The *K_D_* value used for CA200645 was obtained from the saturation‐binding experiments for rat A_2A_ or A_2B_ receptors.

For GloSensor experiments, concentration‐response curves were fitted to the following equation:

$$\text{Response}=\frac{E_{\text{MAX}\;}\times \left[A\right]}{\left[A\right]+{\text{EC}}_{50}},$$

where *E*_MAX_ is the maximum response, [*A*] is the agonist concentration and EC_50_ is the molar concentration of agonist required to generate 50% of the *E*_MAX_. For all statistical analyses, a value of *p* < .05 was considered significant.

## 3 RESULTS

### 3.1 Baseline cardiovascular recordings

**FIGURE 1:**
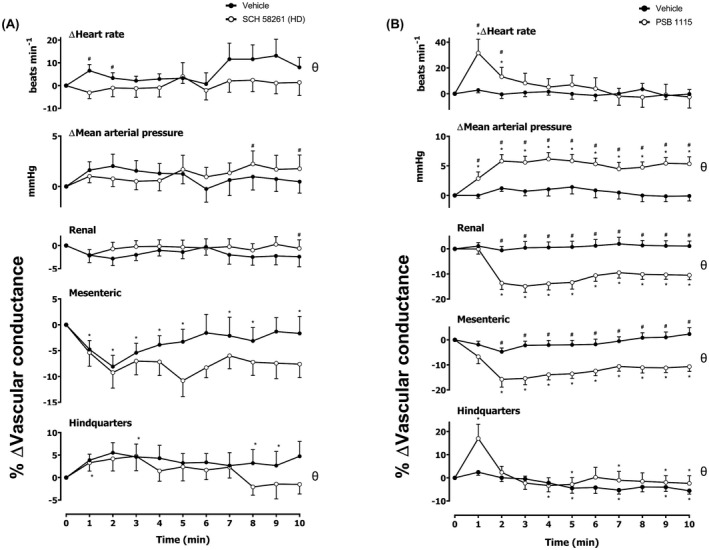
Cardiovascular responses to (A) SCH 58261 or (B) PSB 1115 in conscious, freely moving rats. Rats were dosed with either (A) SCH 58261 (0.1 ml bolus dose of 1 mg kg^−1^ i.v., *n* = 16) or vehicle (0.1 ml bolus dose of 5% propylene glycol, 2% Tween 80 in sterile saline, *n* = 16); or PSB 1115 (0.1 ml bolus dose of 10 mg kg^−1^ i.v., *n* = 16) or vehicle as described in the methods. The time course shows the responses over the 10‐min period, post‐dosing. Data points are mean and vertical bars represent SEM. *p < .05 versus baseline (Friedman's test). A Wilcoxon signed‐rank test was conducted between treated and vehicle control groups for a comparison of area under/over the curve (^θ^ *p* < .05) and to determine differences at each time point (^#^ *p* < .05, Wilcoxon *T*‐test equivalent)

**FIGURE 2:**
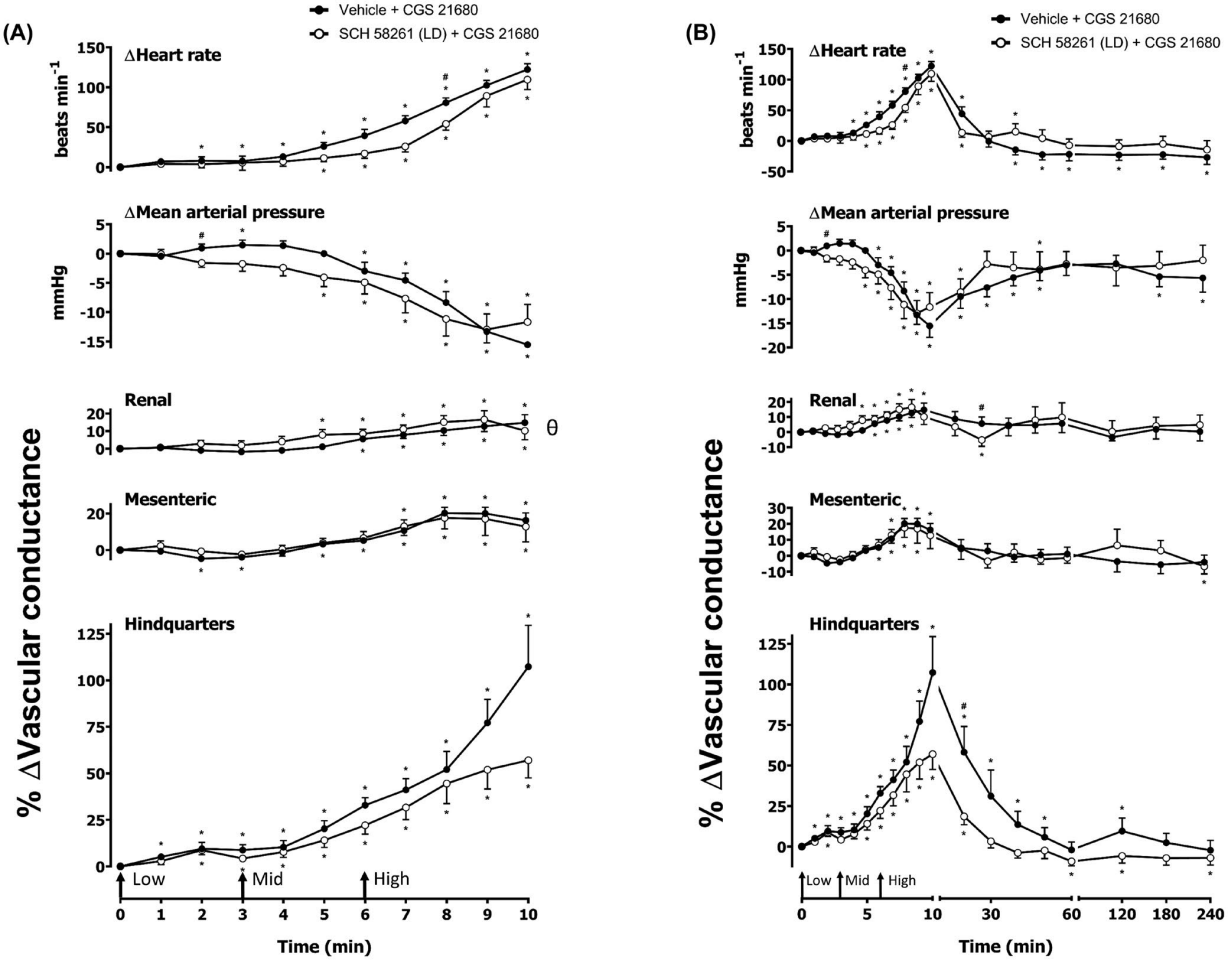
Cardiovascular responses to CGS 21680 in the presence or absence of SCH 58261, in conscious freely moving rats. Rats were dosed with SCH 58261 (0.1 ml bolus dose of 0.1 mg kg^−1^ i.v., *n* = 9) or vehicle (0.1 ml bolus dose of 5% propylene glycol, 2% Tween 80 in sterile saline, *n* = 9) as described in the methods. Approximately 10 min later, all animals received an infusion of CGS 21680 0.1 (Low), 0.3 (Mid) and 1 (high) μg kg^−1^ min^−1^; each dose infused (i.v.) over 3 min (The start of each infusion is indicated by arrows on the *x*‐axis). The time courses show (A) the treatment period and (B) the treatment period plus the extended 4 h recording period. Data points are mean and vertical bars represent SEM. *p < .05 versus baseline (Friedman's test). A Wilcoxon signed‐rank test was conducted between treated and vehicle control groups for a comparison of area under/over the curve (^θ^ *p* < .05) and to determine differences at each time point (^#^ *p* < .05, Wilcoxon *T*‐test equivalent)

**FIGURE 3:**
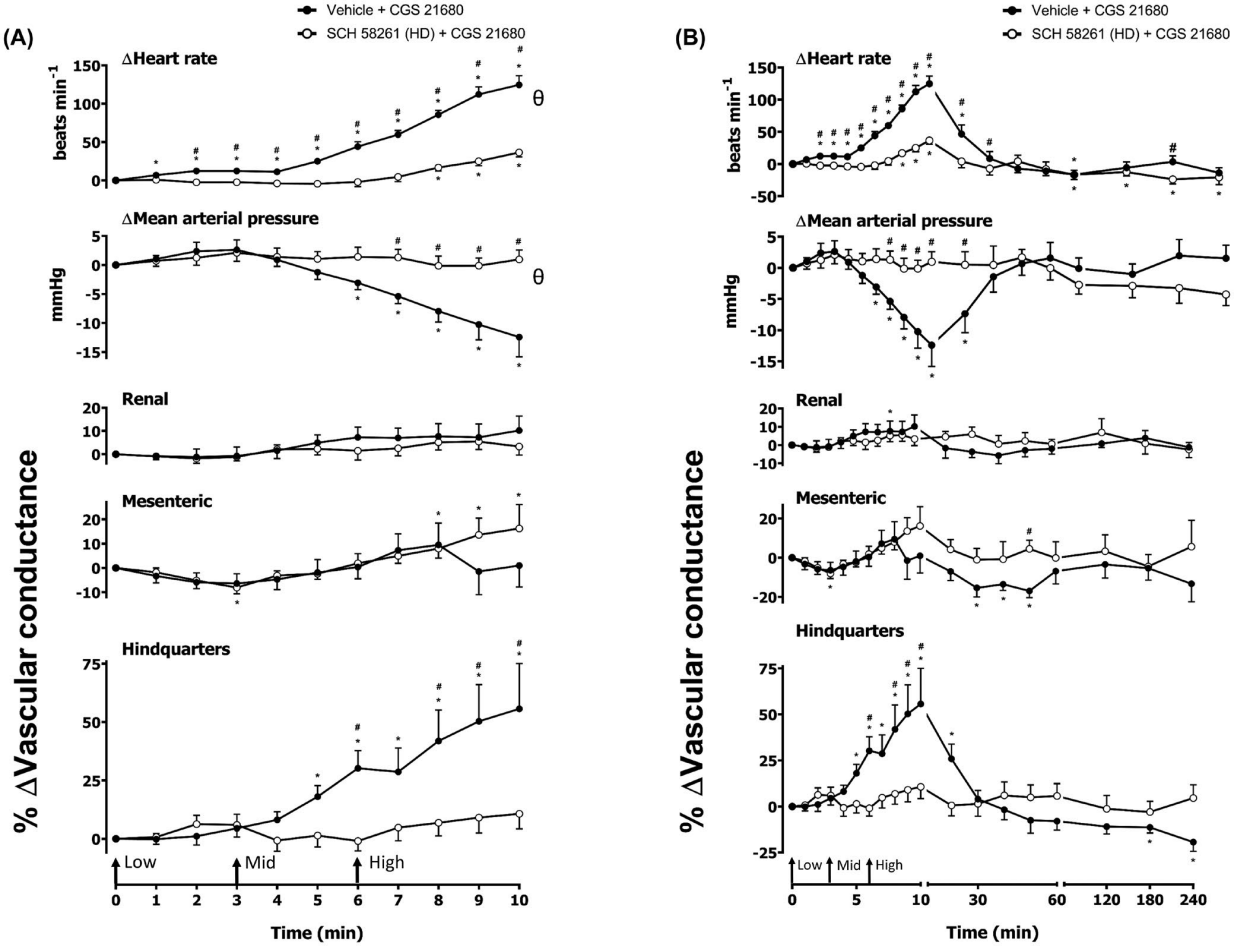
Cardiovascular responses to CGS 21680 in the presence or absence of SCH 58261, in conscious freely moving rats. Rats were dosed with SCH 58261 (0.1 ml bolus dose of 1 mg kg^−1^ i.v., *n* = 8) or vehicle (0.1 ml bolus dose of 5% propylene glycol, 2% Tween 80 in sterile saline, *n* = 8) as described in the methods. Approximately 10 min later, all animals received an infusion of CGS 21680 0.1 (Low), 0.3 (Mid) and 1 (High) μg kg^−1^ min^−1^; each dose infused (i.v.) over 3 min (The start of each infusion is indicated by arrows on the *x*‐axis). The time courses show (A) the treatment period and (B) the treatment period plus the extended 4 h recording period. Data points are mean and vertical bars represent SEM. *p < .05 versus baseline (Friedman's test). A Wilcoxon signed‐rank test was conducted between treated and vehicle control groups for a comparison of area under the curve (^θ^ *p* < .05) and to determine differences at each time point (^#^ *p* < .05, Wilcoxon *T*‐test equivalent)

**FIGURE 4:**
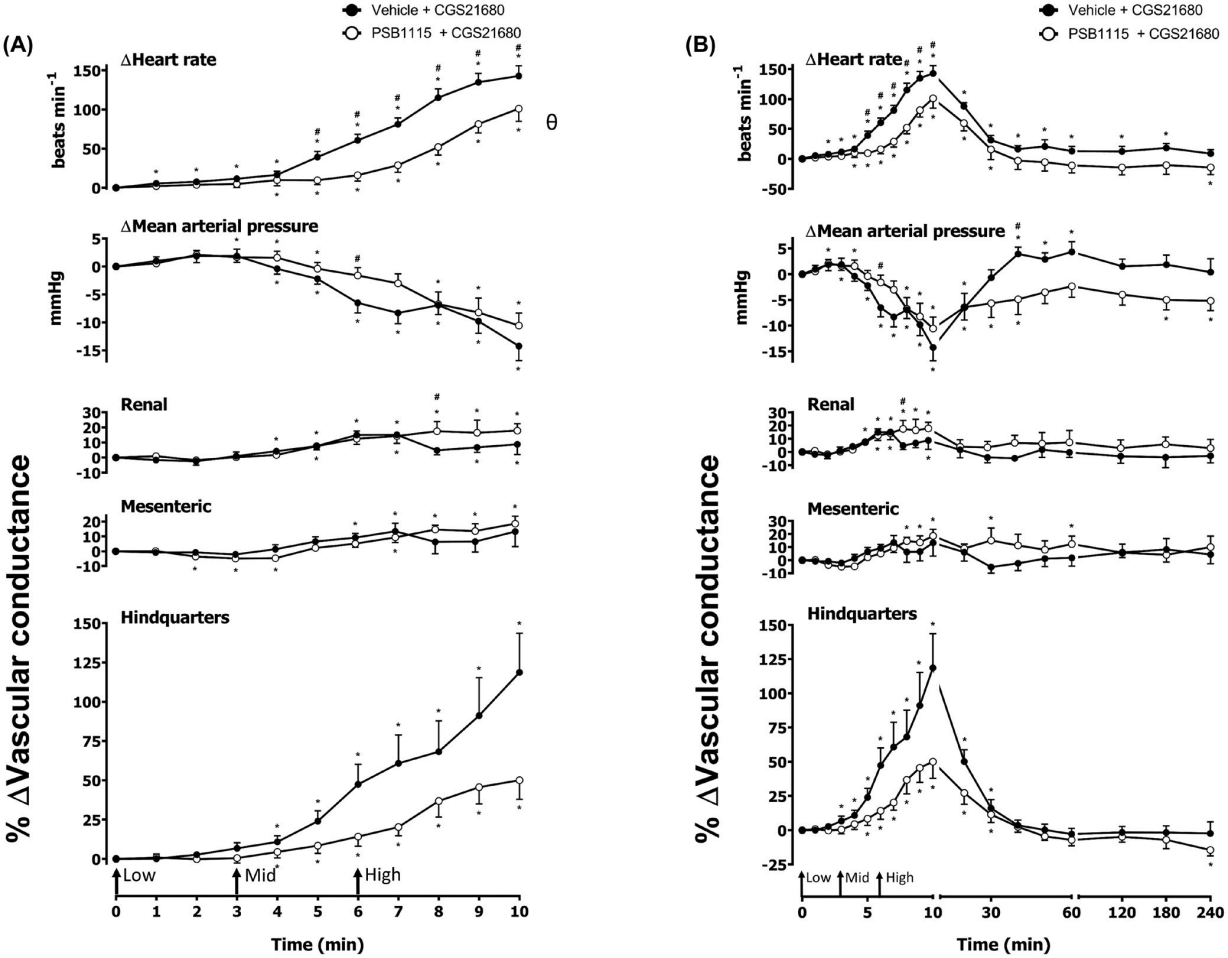
Cardiovascular responses to CGS 21680 in the presence or absence of PSB 1115, in conscious freely moving rats. Rats were dosed with PSB 1115 (0.1 ml bolus dose of 10 mg kg^−1^ i.v., *n* = 8) or vehicle (0.1 ml bolus dose of 5% propylene glycol, 2% Tween 80 in sterile saline, *n* = 8) as described in the methods. Approximately 10 min later, all animals received an infusion of CGS 21680 0.1 (Low), 0.3 (Mid) and 1 (High) μg kg^−1^ min^−1^; each dose infused (i.v.) over 3 min (The start of each infusion is indicated by arrows on the *x*‐axis). The time courses show (A) the treatment period and (B) the treatment period plus the extended 4 h recording period. Data points are mean and vertical bars represent SEM. *p < .05 versus baseline (Friedman's test). A Wilcoxon signed‐rank test was conducted between treated and vehicle control groups for a comparison of area under the curve (^θ^ *p* < .05) and to determine differences at each time point (^#^ *p* < .05, Wilcoxon *T*‐test equivalent)

**FIGURE 5:**
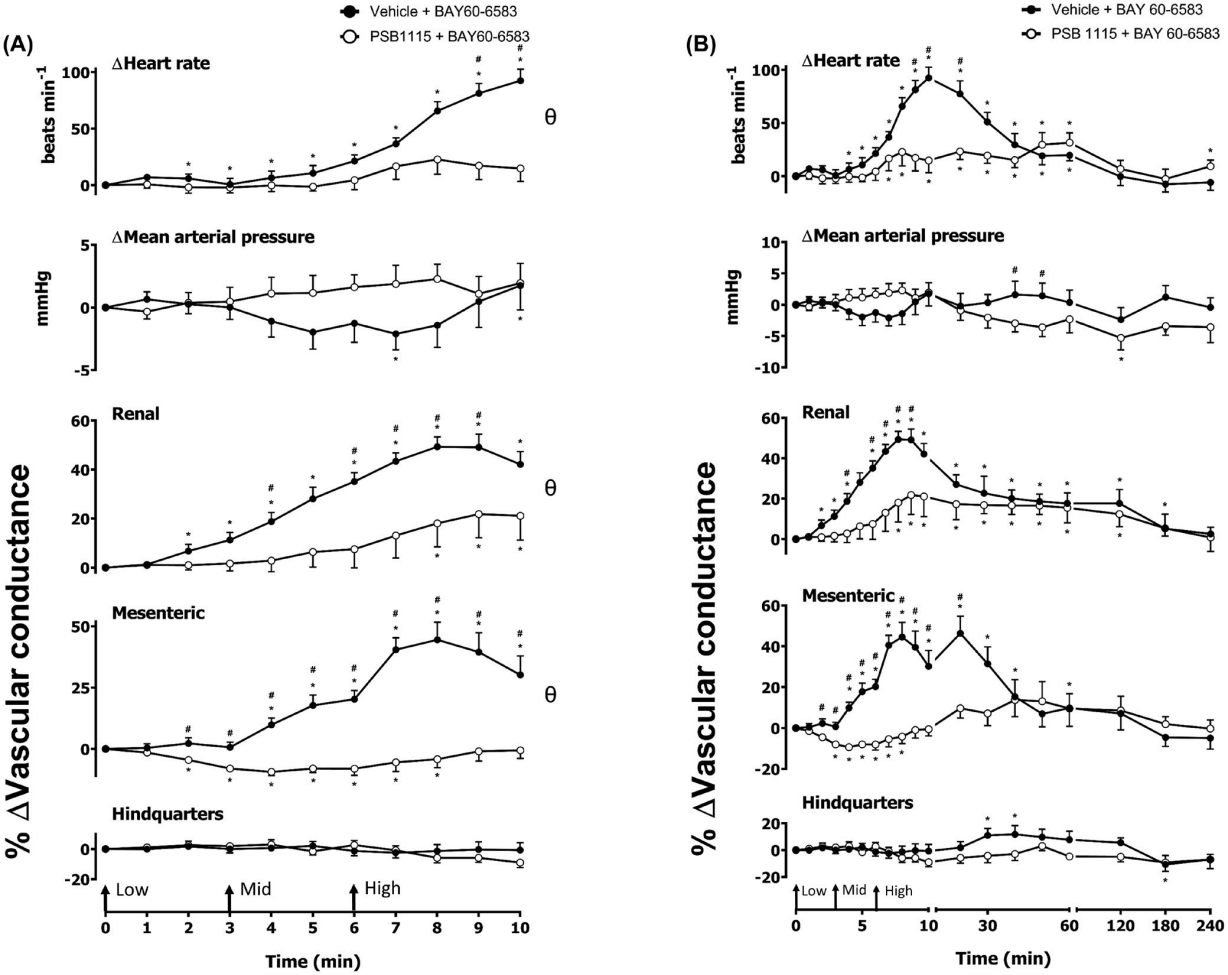
Cardiovascular responses to BAY 60‐6583 in the presence or absence of PSB 1115, in conscious freely moving rats. Rats were dosed with PSB 1115 (0.1 ml bolus dose of 10 mg kg^−1^ i.v., *n* = 8) or vehicle (0.1 ml bolus dose of 5% propylene glycol, 2% Tween 80 in sterile saline, *n* = 8) as described in the methods. Approximately 10 min later, all animals received an infusion of BAY 60‐6583 4 (Low), 13.3 (Mid) and 40 (High) μg kg^−1^ min^−1^; each dose infused (i.v.) over 3 min (The start of each infusion is indicated by arrows on the *x*‐axis). The time courses show (A) the treatment period and (B) the treatment period plus the extended 4 h recording period. Data points are mean and vertical bars represent SEM. *p < .05 versus baseline (Friedman's test). A Wilcoxon signed‐rank test was conducted between treated and vehicle control groups for a comparison of area under the curve (^θ^ *p* < .05) and to determine differences at each time point (^#^ *p* < .05, Wilcoxon *T*‐test equivalent)

**FIGURE 6:**
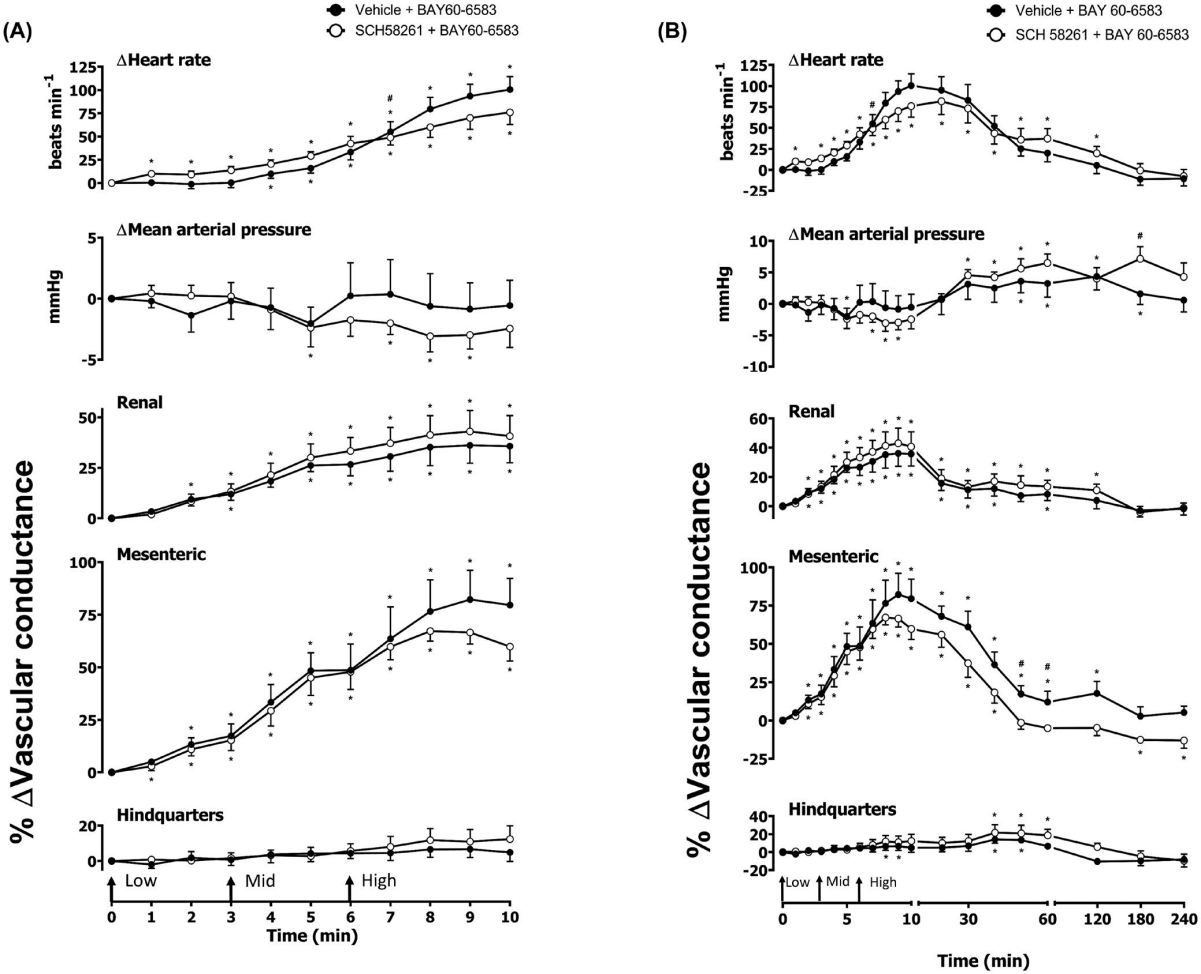
Cardiovascular responses to BAY 60‐6583 in the presence or absence of SCH 58261, in conscious freely moving rats. Rats were dosed with SCH 58261 (0.1 ml bolus dose of 1 mg kg^−1^ i.v., *n* = 9) or vehicle (0.1 ml bolus dose of 5% propylene glycol, 2% Tween 80 in sterile saline, *n* = 9) as described in the methods. Approximately 10 min later, all animals received an infusion of BAY 60‐6583 4 (Low), 13.3 (Mid) and 40 (High) μg kg^−1^ min^−1^; each dose infused (i.v.) over 3 min (The start of each infusion is indicated by arrows on the *x*‐axis). The time courses show (A) the treatment period and (B) the treatment period plus the extended 4 h recording period. Data points are mean and vertical bars represent SEM. *p < .05 versus baseline (Friedman's test). A Wilcoxon signed‐rank test was conducted between treated and vehicle control groups for a comparison of area under the curve (^θ^ *p* < .05) and to determine differences at each time point (^#^ *p* < .05, Wilcoxon *T*‐test equivalent)

For each individual study, baseline measurements taken before the administration of the adenosine receptor agonists SCH 58261 or PSB 1115, adenosine receptor agonists and their corresponding vehicle controls are shown in Table 1. These values correspond to the baselines found in Figures 2, 3, 4, 5, 6. Table 2 presents a summation of baseline results from individual studies before the addition of the adenosine receptor antagonists. These values correspond to the baselines found in Figure 1.

**TABLE 1:** Cardiovascular variables prior to administration of adenosine agonists and antagonists

Baseline *T* = 0	Study 1				Study 2				Study 3				Study 4				Study 5			
	Vehicle		SCH 58261 (LD)		Vehicle		SCH 58261 (HD)		Vehicle		PSB 1115		Vehicle		PSB 1115		Vehicle		SCH 58261 (HD)	
	Mean ± SEM	*n*	Mean ± SEM	*n*	Mean ± SEM	*n*	Mean ± SEM	*n*	Mean ± SEM	*n*	Mean ± SEM	*n*	Mean ± SEM	*n*	Mean ± SEM	*n*	Mean ± SEM	*n*	Mean ± SEM	*n*
Heart rate (beats min^–1^)	353 ± 8	9	344 ± 8	9	345 ± 7	8	356 ± 9	8	342 ± 11	8	353 ± 7	8	350 ± 8	8	362 ± 12	8	343 ± 6	9	357 ± 10	9
Mean BP (mmHg)	107 ± 2	9	104 ± 3	9	96 ± 1	8	97 ± 2	8	101 ± 2	8	100 ± 3	8	105 ± 3	8	105 ± 3	8	105 ± 2	9	108 ± 2	9
Renal VC (U)	70 ± 5	8	77 ± 7	8	72 ± 8	6	70 ± 9	6	98 ± 7	6	113 ± 15	6	74 ± 10	7	80 ± 9	7	85 ± 7	9	89 ± 10	9
Mesenteric VC (U)	89 ± 5	8	91 ± 4	8	74 ± 15	6	88 ± 10	6	95 ± 15	7	104 ± 15	7	91 ± 8	8	89 ± 11	8	72 ± 4	8	78 ± 7	8
Hindquarters VC (U)	41 ± 6	8	48 ± 5	8	48 ± 7	7	46 ± 4	7	45 ± 4	8	46 ± 5	8	46 ± 5	8	48 ± 7	8	44 ± 4	8	42 ± 6	8
**Prior to infusion (*t* = 10 min** ^**†**^ **)**																				
Heart rate (beats min^–1^)	360 ± 9	9	346 ± 10	9	346 ± 8	8	356 ± 8	8	338 ± 11	8	359 ± 20	8	350 ± 9	8	352 ± 13	8	353 ± 9	9	349 ± 9	9
Mean BP (mmHg)	108 ± 2	9	107 ± 3	9	96 ± 1	8	99 ± 2	8	101 ± 2	8	106 ± 4	8	105 ± 2	8	110 ± 3*	8	105 ± 2	9	106 ± 1	9
Renal VC (U)	73 ± 5	8	74 ± 7	8	71 ± 8	6	71 ± 10	6	102 ± 11	6	99 ± 15	6	74 ± 10	7	74 ± 8	7	86 ± 8	9	90 ± 10	9
Mesenteric VC (U)	88 ± 5	8	84 ± 4	8	73 ± 13	6	83 ± 9	6	100 ± 18	7	95 ± 14	7	90 ± 8	8	79 ± 8	8	69 ± 5	8	77 ± 7	8
Hindquarters VC (U)	41 ± 7	8	47 ± 5	8	51 ± 6	7	50 ± 6	7	42 ± 4	8	44 ± 4	8	45 ± 6	8	47 ± 6	8	47 ± 5	8	39 ± 5	8

Notes

Values are mean ± SEM Units of vascular conductance (VC) are kHz. mmHg^–1^ × 10^3^. *N* = 6‐9 per group. Wilcoxson matched‐pairs signed‐rank test.

Abbreviations: U, units; VC, vascular conductance.

* *p* < .05 versus corresponding vehicle group.

† In some instances, agonist administration was delayed past 10 min in the case of movement to allow the rat to settle.

**TABLE 2:** Cardiovascular variables prior to administration of adenosine antagonists for combined study datasets

Baseline *T* = 0	Combination of Studies 2 & 5				Combination of Studies 3 & 4			
	Vehicle		SCH 58261 (HD)		Vehicle		PSB 1115	
	Mean ± SEM	*n*	Mean ± SEM	*n*	Mean ± SEM	*n*	Mean ± SEM	*n*
Heart rate (beats min^–1^)	344 ± 5	17	357 ± 6*	17	346 ± 7	16	358 ± 7	16
Mean BP (mmHg)	101 ± 2	17	103 ± 2	17	103 ± 2	16	102 ± 2	16
Renal VC (U)	80 ± 5	15	81 ± 7	15	85 ± 7	13	95 ± 9	13
Mesenteric VC (U)	73 ± 7	14	82 ± 6	14	93 ± 8	15	96 ± 9	15
Hindquarters VC (U)	46 ± 4	15	44 ± 4	15	46 ± 3	16	47 ± 4	16

Notes

Values are mean ± SEM Units of vascular conductance (VC) are kHz. mmHg^−1^ × 10^3^. *N* = 6–9 per group. Wilcoxson matched‐pairs signed‐rank test.

Abbreviations: U, units; VC, vascular conductance.

* *p* < .05 versus corresponding vehicle group.

### 3.2 The effect of A_2A_ and A_2B_ antagonists SCH 58261 and PSB 1115 on baseline cardiovascular responses

Prior to the administration of all agonists, a bolus of vehicle, SCH 58261 (A_2A_ antagonist), PSB 1115 (A_2B_ antagonist), or respective vehicle control was administered. The effects of the A_2A_ and A_2B_ antagonists were evaluated for 10 min (Figure 1). Significant differences in HR, MAP, renal VC, and hindquarters VC between vehicle and the A_2A_ antagonist, SCH 58261 (1 mg kg^–1^) group were determined by evaluating difference between groups (*p* < .05; area under the curve, AUC) and between groups at given time points; (*p* < .05; Figure 1A). However, no significant changes compared to baseline were observed. Interestingly, however, significant changes in the vehicle mesenteric VC were observed compared to baseline (*p* < .05; Figure 1A). In contrast, the A_2B_ antagonist, PSB 1115 (10 µg kg^−1^) alone caused sustained and significant increases in MAP, accompanied by vascular constrictions in the renal and mesenteric vascular beds (*p* < .05; Figure 1B). These effects may be a result of antagonism of the A_2B_ responses to endogenous adenosine. There was also an initial increase in HR observed; however, this increase was transient and returned to baseline after 3 min (*p* < .05; Figure 1B).

### 3.3 Effect of A_2A_ agonist CGS 21680

Increasing concentrations of CGS 21680 (0.1, 0.3, and 1 µg kg^−1^ min^−1^; 3 min infusions of each dose) were given via intravenous infusion (Figures 2A,B and 3A,B). The hemodynamic profile of CGS 21680 showed a striking increase in HR and hindquarters VC, in addition to moderate increases in renal and mesenteric VCs, which were indicative of vasodilatations (*p* < .05; Figures 2A,B and 3A,B). These effects were accompanied by a significant decrease in MAP (*p* < .05; Figures 2A,B and 3A,B). All parameters returned to baseline approximately 60 min after the final dose of CGS 21680 was administered (Figures 2B and 3B). A 0.1 mg kg^–1^ dose of the A_2A_ antagonist SCH 58261 produced a small attenuation of the hemodynamic responses to CGS 21680 in HR, MAP, renal VC, and hindquarters VC (*p* < .05; Figure 2A,B) with no effect observed in the other vascular beds (Figure 2A,B). In a repeat experiment with a higher dose of 1 mg kg^−1^ of SCH 5861, this antagonist produced a large attenuation of the vasodilator response to CGS 21680 in the hindquarters (*p* < .05), and also inhibited the increase in HR and fall in MAP (*p* < .05; Figure 3A,B). In this experiment, the CGS 21680‐induced changes in renal and mesenteric flow were less pronounced (*p* < .05; Figure 3A,B) and only one time point in the mesenteric VC demonstrated attenuation by SCH 58261 (50 min; *p* < .05; Figure 3B). We also evaluated the effect of the A_2B_‐receptor antagonist PSB 1115 (10 mg kg^–1^) on the cardiovascular responses to CGS 21680 (Figure 4). In this experiment, a similar response to CGS 21680 was observed on HR, MAP, and VC in the three vascular beds (Figure 4A,B). A small inhibition of the HR, MAP, renal VC, and hindquarters VC was observed with PSB 1115, which may be attributable to its affinity for A_2A_ receptors (see below) (*p* < .05; Figure 4A,B). There were, however, no consistent effects of PSB 1115 on the small changes in VC induced by CGS 21680 in the mesenteric arteries (Figure 4A,B).

### 3.4 Effect of A_2B_ agonist BAY 60‐6583

Intravenous infusions of increasing concentrations of BAY 60‐6583 (4, 13.3, and 40 μg kg^−1^ min^−1^; 3 min infusions of each dose) produced dose‐dependent significant increases in HR and VC in the renal and mesenteric vascular beds (*p* < .05; Figure 5A,B). A minor decrease in MAP was also observed during the infusion of the middle (13.3 μg kg^−1^ min^−1^) and highest (40 μg kg^−1^ min^−1^) doses of BAY 60‐6583 (*p* < .05; Figure 5A). MAP returned to baseline immediately after dosing ceased (Figure 5B). No consistent effect was observed in the hindquarters vascular bed during the dosing period (Figure 5A); however, during the 4 h experiment, significant changes from baseline were observed (*p* < .05; Figure 5B). The A_2B_ antagonist PSB 1115 strongly attenuated the BAY 60‐6583 induced HR, MAP and mesenteric VC responses (*p* < .05; Figure 5A,B). However, at the highest dose of BAY 60‐6583 used the increase in renal VC were not fully attenuated by PSB 1115 (*p* < .05; Figure 5A,B). To investigate any involvement of A_2A_‐receptors in the cardiovascular responses to BAY 60‐6583, we also investigate the effect of A_2A_ antagonist SCH 58261 on these responses (Figure 6A,B). In this experiment BAY 60‐6583 produced very similar responses to those reported in Figure 5A,B. However, SCH 58261 (1 mg kg^–1^) only had a small effect on the increase in HR and no significant effect on the increase in VC observed in the renal vascular bed (Figure 6A,B). Interestingly, after the 10 min dosing period, SCH 58261 caused a small attenuation to the BAY 60‐6583 mediated vasodilatations in the mesenteric vascular bed (*p* < .05; Figure 6B).

### 3.5 Measurement of the specific binding of fluorescent adenosine receptor antagonist CA200645 to rat A_2A_ and A_2B_ receptors

**FIGURE 7:**
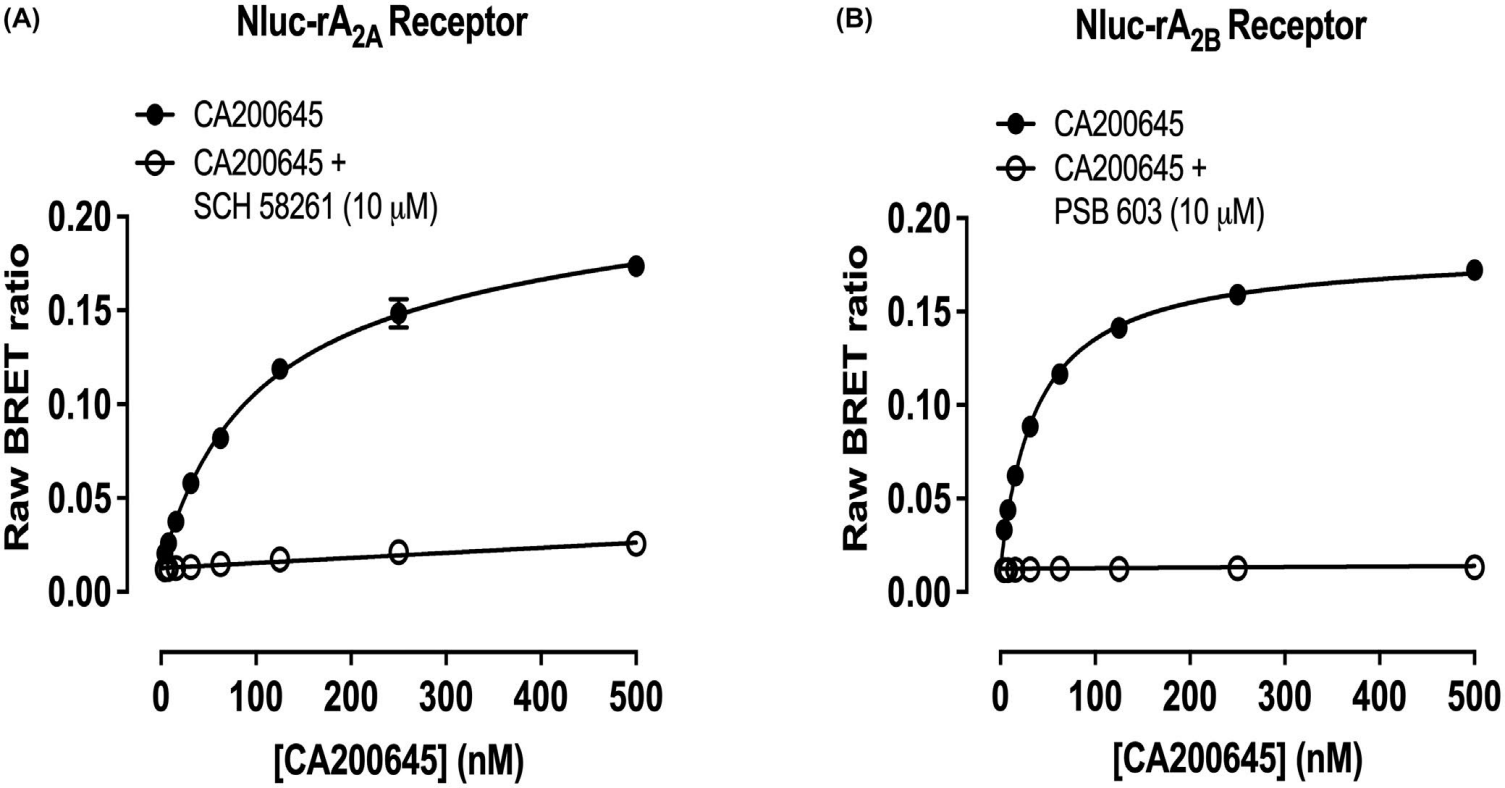
Saturation binding of increasing concentrations of CA200645 to HEK293T cells expressing (A) Nluc‐rat A_2A_ or (B) Nluc‐rat A_2B_, in the presence or absence of (A) 10 µM SCH 58261 or (B) 10 µM PSB 603 (to define non‐specific binding). Data are expressed as raw BRET ratios. Ligands were added simultaneously to triplicate wells and incubated for 120 min at 37°C. Values represent mean ± SEM of five independent experiments

Due to the complexity of whole animal systems, it was necessary to conduct studies to confirm the binding of ligands used in vivo at rat A_2A_ and A_2B_ receptors. Recombinant human A_2A_ and A_2B_ receptors have previously been used to investigate receptor pharmacology in radioligand binding assays in membranes.^26^, ^27^ These assays have provided insight into the selectivity and affinity of A_2A_ and A_2B_ orthosteric ligands. However, more recent technologies such as Nano‐luciferase (Nluc) bioluminescence resonance energy transfer (BRET) have provided advantages over the traditional ligand binding assays.^28^ Therefore, here we have used a NanoBRET proximity assay to assess ligand binding at rat A_2A_ and A_2B_ receptors.^28^, ^29^ To do this, we used N‐terminal Nluc‐tagged A_2A_ and A_2B_ receptors in conjunction with the fluorescent xanthine amine congener‐based antagonist CA200645.^28^, ^29^, ^30^ Saturation analysis demonstrated clear specific binding of CA200645 to Nluc tagged rat A_2A_R and A_2B_R (pK_D_ for CA200645 to: rat Nluc‐A_2A_R = 7.02 ± 0.03, *n* = 5 or rat Nluc‐A_2B_R = 7.43 ± 0.03, *n* = 5) (Table 3; Figure 7). The level of nonspecific binding observed in the presence of 10 µM SCH 58261 for Nluc‐A_2A_R and 10 µM PSB 603 for Nluc‐A_2B_R was low across the concentration range of CA200645 used (Figure 7), confirming that the BRET effects observed during the saturation analysis were specifically caused by binding to the Nluc tagged receptor under investigation.

**TABLE 3:** Binding affinities of six competing ligands determined from inhibition of the specific binding of CA200645 at the rat Nluc‐A_2A_ and Nluc‐A_2B_ receptors expressed in HEK 293T cells

Ligand	Nluc‐rat A_2A_ (pK_D_ or pK_i_)	*n*	Nluc‐rat A_2B_ (pK_D_ or pK_i_)	*n*
CA200645	7.02 ± 0.03	5	7.43 ± 0.03	5
CGS 21680	6.63 ± 0.04	5	n/a	5
SCH 58261	8.67 ± 0.08	5	6.15 ± 0.05	5
BAY 60‐6583	n/a	5	6.33 ± 0.03	5
PSB 1115	5.54 ± 0.03	5	6.27 ± 0.04	5
PSB 603	6.31 ± 0.07	5	8.70 ± 0.03	5
NECA	6.13 ± 0.06	5	5.47 ± 0.07	5

Notes

Data are expressed as mean ± SEM on *n* separate experiments, performed in triplicate. pKi values were determined from IC_50_ values using the Cheng–Prusoff equation. pK_D_ values for CA200645 were determined from saturation binding experiments as described in Figure 7.

### 3.6 Inhibition of CA200645 binding to rat A_2A_ and A_2B_ receptors

**FIGURE 8:**
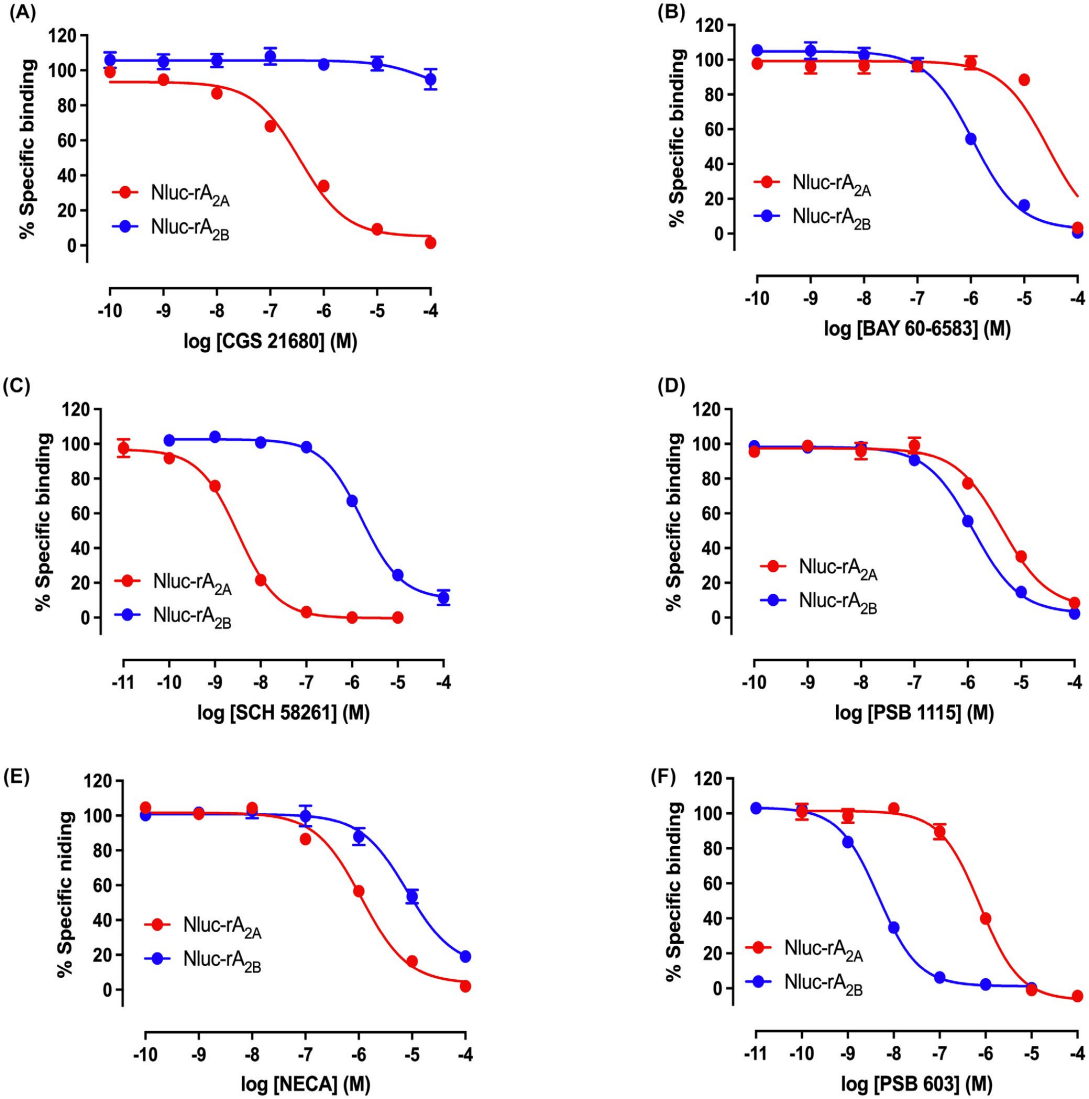
Inhibition of the binding of CA200645 (50 nM) to NanoLuc‐tagged rat‐A_2A_ by A_2A_‐selective ligands (A, C, E) or rat A_2B_ receptors by A_2B_‐selective ligands (B, D, F) by increasing concentrations of competitors. CA200645 and inhibitors were added simultaneously to triplicate wells and incubated for 2 h at 37°C. Values represent mean ± SEM of five independent experiments. Data are expressed as a percentage of the specific binding of 50 nM CA200645 obtained in each experiment. pKi values are shown in Table 3

The binding affinities of a panel of adenosine ligands were determined by competition binding studies in the presence of 50 nM CA200645 (Table 3). Inhibition of specific CA200645 binding to rat Nluc‐A_2A_R or Nluc‐A_2B_R was measured by increasing concentrations of 6 ligands: CGS 21680, NECA, BAY 60‐6583, SCH 58261, PSB 1115, and PSB 603 (Figure 8). K_i_ values obtained from these studies indicated that CGS 21680 was selective for Nluc‐A_2A_R over Nluc‐A_2B_R, whereas BAY 60‐6583 was selective for the Nluc‐A_2B_R at concentrations up to 10 µM (Table 3) (Figure 8). At 100 µM BAY 60‐6583, there was a marked attenuation of binding to Nluc‐A_2A_R. SCH 58261, PSB 1115, and PSB 603 all displaced specific CA200645 binding to both Nluc‐A_2A_R and Nluc‐A_2B_R (Figure 8). As expected, SCH 58261 had a much higher affinity for Nluc‐A_2A_R than Nluc‐A_2B_R, and PSB 603 and PSB 1115 had higher affinities for Nluc‐A_2B_R over Nluc‐A_2A_R.

### 3.7 Effect of BAY 60‐6583 on functional responses to CGS 21680 and formoterol

**FIGURE 9:**
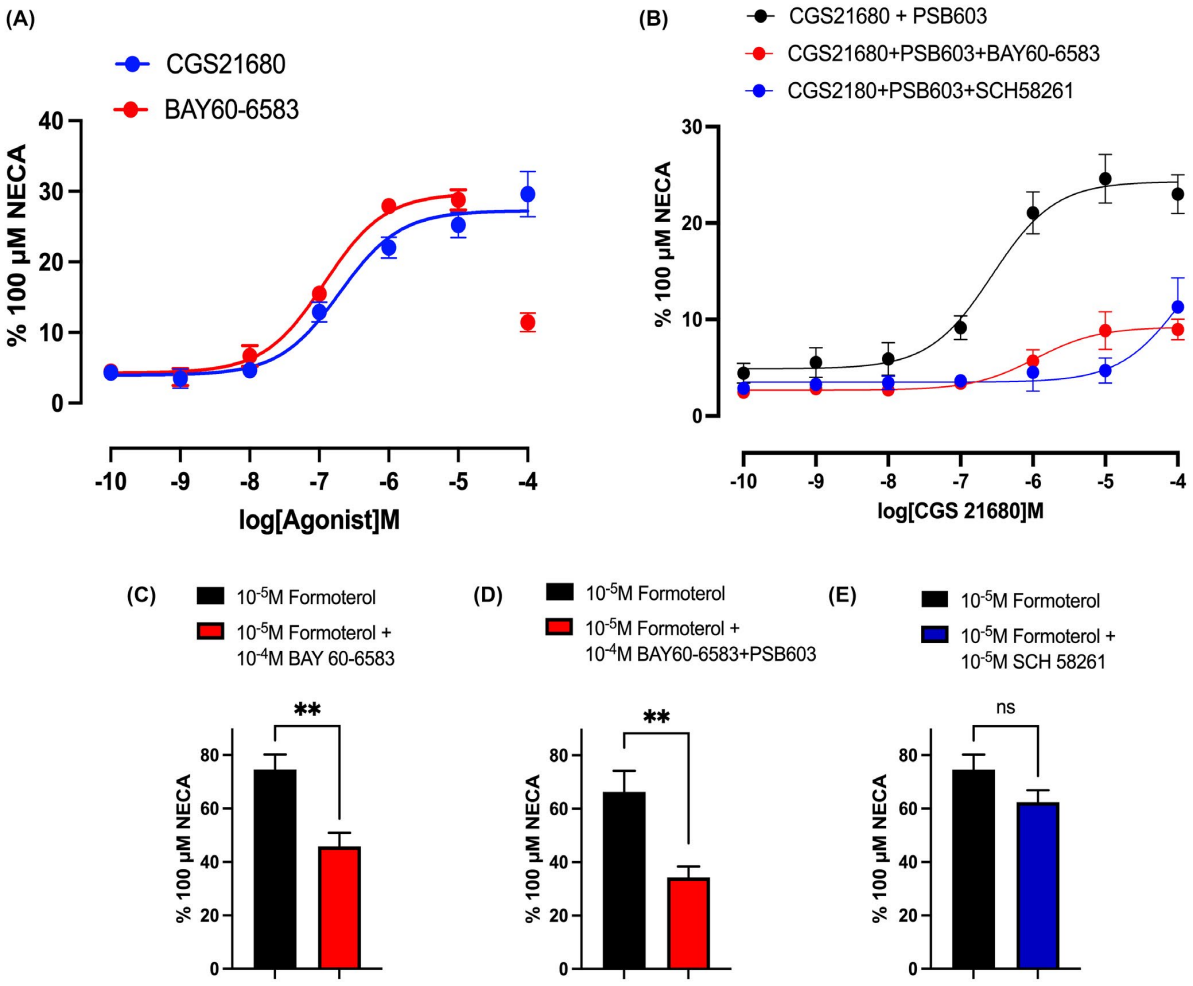
Glosensor cyclic AMP responses to CGS 21680 and BAY 60‐6583 in HEK293G cells. (A) Concentration‐response curves for agonist‐stimulated cAMP Glosensor luminescence responses in HEK293G cells in response to CGS 21680 and BAY 60‐6583. (B) The effect of simultaneous addition of 100 µM BAY 60‐6583 on the concentration‐response curve to CGS 21680, following 2 h pretreatment with PSB 603 (0.1 µM). Also shown is the effect of simultaneous addition of 10 µM SCH 58261 on the responses to CGS 21680 in the presence of PSB 603 (0.1 µM). (C, D) The effect of 100 µM BAY 60‐6583 on the response to 10 µM formoterol in the (C) absence and (D) presence of 0.1 µM PSB 603. (E) The effect of simultaneous addition of 10 µM SCH 58261 on the response to 10 µM formoterol. Data represent mean ± SEM of the peak luminescence response of 5 separate experiments, each carried out in triplicate. Data are expressed as a percentage of the peak luminescence response obtained with 100 µM NECA measured in the same experiment. **p < .05 (unpaired *t*‐test)

To further evaluate the effect of BAY 60‐6583 at high concentrations, we investigated its effect on agonist responses to endogenous A_2A_, A_2B_, and β_2_‐adrenoceptors expressed in native HEK293 cells expressing the Glosensor cyclic AMP biosensor (HEK293G).^14^ CGS 21680 and BAY 60‐6583 elicit cyclic AMP responses that are selectively mediated via A_2A_ and A_2B_ receptors respectively.^14^ In our hands, both CGS 21680 and BAY 60‐6583 elicited responses similar to those reported previously (Figure 9).^14^ However, it was notable that at the highest concentration employed (100 µM), there was a marked drop in the response to BAY 60‐6583 (Figure 9A). In the presence of the A_2B_‐selective antagonist PSB 603, the response to the A_2A_ agonist CGS 21680 (mediated via A_2A_ receptors) was significantly attenuated by simultaneous treatment with BAY 60‐6583 (Figure 9B). However, this appeared to be a nonspecific effect since 100 µM BAY 60‐6583 similarly affected responses to the β_2_‐agonist formoterol in the same cells (Figure 9C,D). The selective A_2A_‐receptor antagonist SCH 58261 (10µM), however, had no significant effect on the response to formoterol (Figure 9E).

## 4 DISCUSSION

In the present study, we set out to evaluate the effect of selective activation of A_2A_ and A_2B_ adenosine receptors on VC in three different vascular beds (renal, mesenteric and hindquarters) of conscious freely moving rats. Previous work in ex vivo models (including those from genetically modified mice lacking either the A_2A_ or A_2B_ receptors) has confirmed that both A_2A_ and A_2B_ receptors can produce vasodilatation in different vascular beds, including the kidney and coronary circulations.^16^, ^17^, ^18^ A_2A_ knock‐out mice are also known to be hypertensive.^7^ We previously used a Doppler flow model to show that adenosine can elicit vasodilatations in the renal, mesenteric, and hindquarters’ vascular beds of conscious rats that is not attenuated by the selective A_1_‐receptor antagonist DPCPX and is likely to be mediated by A_2_ receptors.^19^ In this study, we used selective agonists for the A_2A_‐ (CGS 21680)^14^ and A_2B_‐ (BAY 60‐6583)^14^, ^20^ receptors to investigate the individual hemodynamic effects of A_2A_ and A_2B_ receptor stimulation on VC.

To investigate the receptor subtype selectivity of the ligands used in vivo for rat adenosine A_2A_ and A_2B_ receptors, we employed a NanoBRET ligand binding study using N‐terminal nanoluciferase‐tagged receptors and the fluorescent ligand CA200645. CA200645 is a fluorescent analog based on the nonselective adenosine receptor antagonist xanthine amine congener.^28^, ^29^, ^30^ It demonstrated saturable specific binding to each receptor and had moderate affinity for the rat A_2A_ (PK_D_ 7.02) and A_2B_ (PK_D_ 7.43) receptors (Table 3). Binding of CA200645 to the relevant receptor was completely displaced by the A_2A_ or A_2B_‐selective antagonists SCH 58261 and PSB 603, respectively. Furthermore, binding of CA200645 to each rat receptor showed the expected selectivity for known A_2A_ and A_2B_‐receptor ligands.

CGS 21680 had a good affinity for the rat Nluc‐tagged A_2A_‐receptor (Table 3; pKi 6.63) and was not able to bind to rat A_2B_‐receptors expressed in HEK293T cells at concentrations up to 100µM. This A_2A_‐selective agonist produced a striking increase in HR and hindquarters VC that was accompanied by a significant decrease in MAP in conscious rats. This was attenuated by the A_2A_‐selective antagonist SCH 58261. Small and less consistent increases in VC were also seen in the renal and mesenteric vascular beds. The renal response was attenuated by SCH 58261, while that in the mesenteric circulation was only marginally affected by this antagonist. In the presence of PSB 1115, the hindquarters response to CGS 21680 was partially antagonized by this A_2B_ antagonist. However, comparison of the binding affinity of PSB 1115 for the rat A_2A_ and A_2B_ receptors measured using NanoBRET confirmed that it has limited selectivity for the A_2B_ receptor (Table 3; pKi 6.13 for A_2B_; 5.54 for A_2A_). It therefore seems likely that any antagonism of the hindquarters’ VC response to CGS 21680 is due to an effect of PSB 1115 on A_2A_ receptors. Unfortunately, solubility issues prevented us from using the more A_2B_ selective antagonist PSB 603 for in vivo experiments.

BAY 60‐6583 had a good affinity for the rat Nluc‐tagged A_2B_‐receptor (Table 3; pKi 6.33) and did not affect A_2A_ binding of the fluorescent ligand CA200645 at concentrations up to 10 µM. However, at 100 µM, BAY 60‐6583 completely attenuated the binding of 50 nM CA200645 to the rat A_2A_ receptor. This is likely due to a non‐specific effect since this high concentration of BAY 60‐6583 also attenuated Glosensor cyclic AMP responses to both CGS 21680 and the β_2_‐agonist formoterol in HEK293G cells. In vivo, 4, 13.3, and 40 µg kg^−1^ min^−1^ doses of BAY 60‐6583 significantly increased HR and VC in the renal and mesenteric vascular bed, but not in the hindquarters. There was little change in MAP. These effects of BAY 60‐6583 were antagonized by the A_2B_‐antagonist PSB 1115, but only to a small extent by the A_2A_‐selective antagonist SCH 58261 (Table 3; pKi A_2A_ 8.67; A_2B_ 6.15), confirming that these effects were mediated by A_2B_‐receptors.

Taken together, these data indicate that A_2A_ and A_2B_ receptors are regionally selective in their regulation of vascular tone. Thus, A_2A_‐receptors mediate a large vasodilatation in the hindquarters with smaller, more limited effects on VC in the renal and mesenteric vascular beds. In contrast, A_2B_ receptor stimulation leads to a large increase in VC in both the renal and mesenteric circulations with no effect on the hindquarters. Interestingly, selective activation of vasodilator responses to A_2A_ or A_2B_ receptor activation both caused a strong increase in HR, but only the A_2A_ effect in the hindquarters is accompanied by a fall in MAP. The tachycardia in the heart following A_2A_ receptor activation is most likely due to a baroreflex excitation of the sympathetic nervous system,^31^, ^32^ and there is also evidence of cardiovascular regulation by A_2B_ receptors in the posterior hypothalamus of the rat.^33^

A striking feature of the selective activation of A_2B_ receptors with BAY 60‐6583 is the marked increase observed in VC in the renal and mesenteric circulation, with only a minor effect on the VC in the hindquarters and MAP. Adenosine has been previously observed to cause vasodilatation in the renal circulation,^19^, ^34^, ^35^ and this has generally been considered to mediate this vasodilator effect via the low‐affinity A_2B_ receptor.^36^ However, studies in the renal microcirculation have also reported expression of the adenosine A_2A_ receptor, which can elicit afferent arteriolar vasodilation by activating K_ATP_ channels.^36^ However, there was no consistent effect of the A_2A_ agonist CGS 21680 in the renal or mesenteric circulations in our studies. These data suggest that the A_2B_ receptor is primarily responsible for an increase in VC in the renal vasculature.

A major advantage of the Doppler flowmetry model is that it can monitor multiple cardiovascular changes in a conscious rat, where compensatory reflex mechanisms are still operational. This explains why marked increases in HR are observed with both A_2A_ and A_2B_ receptor stimulation and, as mentioned earlier, these are likely to be a result of baroreflex excitation of the sympathetic nervous system in the posterior hypothalamus.^31^, ^32^, ^33^ Consistent with this effect is the pronounced A_2A_‐mediated vasodilatation in the hindquarters that is matched with both an increase in HR and a fall in MAP. More surprising was that large increases in VC in the renal and mesenteric circulations induced by A_2B_ receptor activation were not accompanied by a change in MAP. In the kidney, A_2B_ and A_2A_ receptors are located in the renal vasculature and cause renovascular dilatation and increased renin secretion.^37^, ^38^, ^39^ This increase in renin release and the subsequent vasoconstrictor action of angiotensin II, combined with a reflex increase in HR, may counteract any changes in MAP following selective A_2B_ receptor activation.

The A_2B_ receptor has been demonstrated to play an important role in renal ischemic reperfusion injury by improving post‐ischemic renal peritubular capillary flow.^39^, ^40^ Thus, the renal protective effect of A_2B_ receptor activation is abolished in A_2B_ receptor knock‐out mice, and BAY 60‐6583 treatment can dramatically improve renal function in wild‐type mice.^40^ Similar results have been obtained in A_2B_ receptor knock‐out mice and with BAY 60‐6583 for mesenteric ischemia.^41^ The results obtained in the present study suggest that A_2B_‐receptor activation produces a vasodilatation in the rat that is selective to particular vascular beds (including the renal circulation) and is not accompanied by changes in blood pressure. This result suggests that the development of A_2B_ receptor agonists to induce vasodilatation in the kidney and mesentery may provide a good therapeutic approach for the treatment of acute kidney injury and mesenteric ischemia.^39^, ^41^

A_2A_‐receptor activation plays a major role in suppressing immune responses,^42^, ^43^, ^44^ and indeed a number of A_2A_‐receptor antagonists are in clinical trials for the treatment of cancer because of their ability to prevent adenosine‐mediated suppression of immune responses in cancer.^45^, ^46^ Thus, stimulating A_2B_‐receptors in acute kidney injury and mesenteric ischemia has potential to avoid some of the immunosuppressive actions of A_2A_‐receptor agonists. However, it remains possible that A_2B_‐receptor activation may also contribute to the suppression of the immune system by adenosine, and dual A_2A_/A_2B_ receptor antagonists are currently undergoing clinical trials in cancer.^46^

## DISCLOSURES

The authors declare no conflicts of interest.

## AUTHOR CONTRIBUTIONS

*Participated in the research design*: Samantha L. Cooper, Edward S. Wragg, Jeanette Woolard, and Stephen J. Hill. *Performed the molecular biology*: Mark Soave and Edward S. Wragg. *Conducted the experiments*: Samantha L. Cooper, Edward S. Wragg, and Patrizia Pannucci. *Performed the data analysis*: Samantha L. Cooper, Edward S. Wragg, Stephen J. Hill, and Jeanette Woolard. *Wrote or contributed to the writing of the manuscript*: Samantha L. Cooper, Edward S. Wragg, Mark Soave, Patrizia Pannucci, Stephen J. Hill, and Jeanette Woolard.

## Data Availability

The data that support the findings of this study are available from the corresponding author upon reasonable request.
